# Toward healthy behavior: fear of missing out, smartphone, social networking addiction, and life satisfaction among medical students at Tanta University, Egypt

**DOI:** 10.1007/s44192-025-00227-0

**Published:** 2025-07-17

**Authors:** Eman Ali Younis, Asmaa Mohammad Ahmad Mohammad, Aya Farag Attia Elsebaey

**Affiliations:** 1 Department of Public Health and Community Medicine Department, Faculty of Medicine, Tanta University, Tanta, 31257 Egypt; 2 Public Health and Community Medicine Department, Faculty of Medicine, Tanta University, Tanta, Egypt

**Keywords:** Fear of missing out, Smartphone, Social networking, Addiction, Life satisfaction, Medical students

## Abstract

**Background:**

The global shift in culture towards “digitalization,” particularly among youth, has made individuals increasingly vulnerable to the behavioral effects of social networking sites and smartphones. This study aims to measure the degree of smartphone and social networking addiction, as well as fear of missing out (FOMO), among medical students and to investigate the relationship between these constructs and life satisfaction.

**Methods:**

A cross-sectional study was conducted at the Faculty of Medicine, Tanta University, between December 2023 and March 2024. A total of 630 students were selected by a simple random technique. Data were collected using a structured self-administered questionnaire that involved the FOMO, smartphone and social media addiction, and life satisfaction scales. The chi-square test was used for categorical variables. Pearson correlation was employed to examine relationships between different scales.

**Results:**

The prevalence of FOMO among medical students is 57.8% moderate and 2.5% high grade. Sixty-three percent are moderate, and 13.2% are high social media addicts. Seventy percent are moderate, and 10.4% are high-level smartphone addicts. FOMO is significantly positively correlated with both smartphone addiction (*p* < 0.001, rs = 0.393) and social media addiction (*p* < 0.00, rs = 0.304). FOMO shows a slight but significant negative correlation with life satisfaction (*p* = 0.027, rs = −0.088). Smartphone addiction has a moderate positive correlation with social media addiction (*p* < 0.00, rs = 0.542).

**Conclusion:**

The prevalence of FOMO among medical students was an alarming sign. Also, social media and smartphone addiction have been becoming issues of concern. The findings of the study showed that FOMO is significantly positively correlated with both smartphone addiction and social media addiction. Smartphone addiction has a moderate positive correlation with social media addiction. FOMO shows a small but significant negative correlation with life satisfaction.

## Introduction

Social media has become an integral part of our lives as a result of technological advancements. Social media networks have made it possible for people to instantly learn about what other people are up to. Additionally, the person can update others on what is going on in his life at that particular time. However, unhealthy use for socialization might create the conditions to suffer from fear of missing out (FOMO) [1].

FOMO is defined as “a pervasive apprehension that others might be having rewarding experiences from which one is absent.” This anxiety creates an environment for the individual to stay in touch and communicate with their social environment in order not to miss anything out. The individual constantly desires to be informed about what others are doing now and is therefore afraid of missing out on developments [2]. FOMO in individuals may be an indicator of a disruption in their mental health and may thus require professional help from a counselor or psychotherapist [3].

FOMO was found to be associated negatively with life satisfaction. As the FOMO level increases, life satisfaction decreases [4]. Using a smartphone problematically and social media addiction may be associated with increased levels of FOMO [5]. A study in the Middle East region in 2020 showed varied university students’ FOMO scores. For instance, students from Pakistan, Jordan, and Egypt scored higher on FOMO than students from the United Arab Emirates or India. Actually, a variety of factors could have had a role in this result, including sociocultural and economic issues, acculturative stress, and others [6].

According to the Global Statshot Report 2024, there were 5.61 billion mobile phone users and 5.04 billion active social media users globally [7]. Social media platforms are utilized by medical students globally for both personal and educational purposes. Social networking tools significantly impacted the learning process and progressively gained insight into education. With more time spent on interactive tools and social media following the COVID-19 pandemic, this contact has reached its peak [8].

A study conducted among medical students found that the majority of them (89.1%) had a presence on social networking sites, and 69.3% believed that it is beneficial for learning. The most common websites used by students for their learning were YouTube (92.1%) and WhatsApp (71.2%) for easy communication. Most students (90.6%) used it for learning for at least 1 h per day or more [9].

University students, particularly medical ones, were chosen as the study’s target demographic. Research has shown their vulnerability to addiction and mental health issues due to their academic stress. Moreover, in collectivistic cultures, there is a greater incidence of FOMO [10].

Future physicians are today’s medical students; a social media addiction and FOMO will impede both their learning process and ability to do daily activities, and their professional vision may be affected by this discomfort [11]. A 2023 Achinese study among medical students showed that learning burnout is significantly impacted by FOMO and that this effect is mediated by smartphone addiction and sleep quality. More significantly, learning burnout may impact medical students’ professional growth and increase dropout rates in addition to negatively affecting their academic performance and general well-being [12].

FOMO is not given enough consideration, despite being a manageable issue. It is anticipated that this study will raise awareness of it and its connection to life satisfaction among psychologists, behaviorists, parents, and caregivers in order to implement coping mechanisms. The current study aims to (1) measure the prevalence of FOMO, social media addiction, and smartphone addiction among medical students at Tanta University; and (2) investigate the interrelationship between these constructs and their association with life satisfaction.

## Methods

### Design, setting, and duration of the study

The survey data for this study were collected in a cross-sectional design from the beginning of December 2023 to the end of March 2024 at the Faculty of Medicine, Tanta University, located in Tanta City, the capital of Al Gharbia Governorate in Egypt’s Nile Delta region. As a central public university in the Mid Delta area, it attracts students from lower Egyptian governorates. The target population consisted of medical students enrolled in the first through fifth academic years.

### Study population and sample size

The Faculty of Medicine had a total of 6000 students across the five academic years. To evaluate Fear of Missing out (FOMO) scores, sample size calculations were based on a prior study by Sabir et al. [13], which indicated that 48.8% of participants had high FOMO scores. To achieve a 95% confidence level with a 5% margin of error and 80% study power, a minimum sample size of 384 was required. Using Epi Info (version 7.2.5.0) software for sample size calculations. We added 20% to compensate for incomplete questionnaires, bringing the total sample size to 460. Researchers distributed 650 questionnaires; 643 were returned and 13 were excluded for missing data, and 630 questionnaires were valid with a response rate of 97%.

### Inclusion and exclusion criteria

The studied participants were selected according to certain inclusion criteria. All students of both genders enrolled in the medical program in the Faculty of Medicine at Tanta University from the first to fifth academic years during the study period who have provided informed consent to participate in the study, excluding medical interns.

### Sampling technique

Students were selected using a simple random sampling technique. The selection process followed these steps: a complete list of all eligible students (based on inclusion criteria) was compiled, ensuring that no student was excluded unfairly. This list was generated using student records, ensuring that the sample represented the full range of the medical student population. A random selection was then performed, typically using a random number generator (a computer program) to choose participants. Once the participants were randomly selected, they were approached individually to invite them to participate in the study after finishing their lecture schedule by data collectors. Researchers distributed 650 questionnaires; 643 were returned and 13 were excluded for missing data, and 630 questionnaires were valid with a response rate of 97%.

### Study tools

Data were collected using a structured questionnaire developed by the authors after reviewing relevant literature [2, 14–16]. The questionnaire included five sections with a total of 37 questions, focusing on socio-demographic data, FOMO, smartphone addiction, social media addiction, and life satisfaction.

The first section covered participants’ age, gender, residence (urban or rural), academic year, daily social media usage (less than 2 h, 2–4 h, more than 4 h), and involvement in hobbies or sports.

The second section covered Fear of Missing Out (FOMO). The FOMO scale featured 10 items rated on a 5-point Likert scale, ranging from 1 (not at all true of me) to 5 (extremely true of me). Scores were categorized as follows: low (10–<25), moderate (25–37), and high (38–50).

The third section covered smartphone addiction: The Chinese Smartphone Addiction Scale—Short Version included 10 items rated from 1 (strongly disagree) to 5 (strongly agree). Scores were divided into low (10–<25), moderate (25–37), and high (38–50).

The fourth section covered social media addiction. The Bergen Social Media Addiction Scale (BSMAS) comprised 6 items rated from 1 (very rarely) to 5 (very often). The scores ranged from 6 to 30 and were categorized as low (6–14), moderate (15–22), and high (23–30).

The fifth section covered Life Satisfaction: The Satisfaction with Life Scale contained 5 items rated on a 7-point Likert scale, ranging from 1 (strongly disagree) to 7 (strongly agree). Scores ranged from 5 to 35 and were classified into low satisfaction (7–17), moderate satisfaction (18–26), and high satisfaction (27–35).

### Validity and reliability of the study tools

According to Przybylski et al. [2], the FOMO scale has a good internal consistency, with a Cronbach alpha coefficient of 0.89. According to Luk et al. [15], smartphone scale has a good internal consistency, with a Cronbach alpha coefficient of 0.844. According to Zarate et al. [16], the social media addiction scale has a good internal consistency. Cronbach’s α = 0.88. According to Diener et al. [14], the life satisfaction scale has a good internal consistency (Cronbach’s α = 0.87).

In our study, the questionnaire’s validity was assessed by five Egyptian consultants from the public health department, who recommended simplifying some questions. A pilot study was conducted with 20 medical students, not included in the final analysis, to test the questionnaire’s reliability. The internal consistency was measured using Cronbach’s alpha for each scale (Fear of Missing Out, Smartphone Addiction, Social Media Addiction, Life Satisfaction), yielding values of 0.83, 0.81, 0.79, and 0.80, respectively, indicating adequate reliability.

### Data collection

Data were collected using a self-administered English-language questionnaire during scheduled lecture times for each academic year, and the average time required for filling out the questionnaire by each student was 10–15 min. Fifth-semester medical students helped the researchers in the data collection stage, who were trained on data collection by the main investigators, to ensure they understood the study’s objectives, ethical considerations, and the proper procedure for data collection and to explain any unclear word or expression in the questionnaire to ensure that all participants have completed the data.

### Statistical analysis

The authors tested the data normality using Shapiro–Wilk and Kolmogorov–Smirnov tests, and the results indicated that the *P* value exceeded 0.05 (not significant). Thus, data analysis Involved calculating means and standard deviations for continuous variables and tested by one-way ANOVA. Number and percentage for categorical variables and tested by Chi-square tests. Pearson correlation was employed to examine relationships between FOMO, smartphone addiction, social media addiction, and life satisfaction. Statistical significance was determined using two-sided *p*-values ≤0.05. All statistical analyses were performed using IBM SPSS Statistics (version 25).

Overall percentage scores for the students for all parts of the questionnaire were then calculated. A total score of less than 50% was rated as low, 50–75% was moderate, and >75% was considered as high [17].

### Ethical considerations

The study followed ethical standards by obtaining informed consent from all students, ensuring the confidentiality of responses, and clarifying that the data would be used solely for research purposes. Ethical approval was granted by the Internal Review Board of the Faculty of Medicine, Tanta University, with code number 36264PR873/24.

## Results

The descriptive analysis showed that the sample consisted of 630 medical students with a mean age of 20.69 ± 1.4 years and slightly more females (59.7%) than males (40.3%). A total of 60.5% of the students were urban. One-fifth (20.5%) were in their final graduation years (fourth and fifth), and 45.7% were in the third year. A considerable percentage (61.3%) had various hobbies and sports. Regarding the number of hours spent using social media per day, 38.4 and 44.6% spent 2–4 and more than 4 h, respectively (Table 1).

**Table 1:** The relationship between sociodemographic data and FOMO levels among medical students

Sociodemographic data			FOMO Levels			Total N = 630	*P* value
			Low n = 250(39.7%)	Moderate n = 364(57.8%)	High n = 16(2.5%)		
Gender	Male	n	96	152	6	254	0.688^(1)^
		%	38.4	41.8	37.5	40.3	
	Female	n	154	212	10	376	
		%	61.6	58.2	62.5	59.7	
Residence	Urban	n	150	222	9	381	0.912^(1)^
		%	60.0	61.0	56.3	60.5	
	Rural	n	100	142	7	249	
		%	40.0	39.0	43.7	39.5	
Academic year	First	n	41	41	2	84	0.333^(1)^
		%	16.4	11.3	12.5	13.3	
	Second	n	51	76	2	129	
		%	20.4	20.9	12.5	20.5	
	Third	n	103	179	6	288	
		%	41.2	49.2	37.5	45.7	
	Fourth	n	36	42	4	82	
		%	14.4	11.5	25.0	13.0	
	Fifth	n	19	26	2	47	
		%	7.6	7.1	12.5	7.5	
Hobbies and sports	No	n	104	134	6	244	0.486^(1)^
		%	41.6	36.8	37.5	38.7	
	Yes	n	146	230	10	386	
		%	58.4	63.2	62.5	61.3	
Number of hours using social media per day	Less than 2 h	n	51	56	0	107	<0.001*^(1)^
		%	20.4	15.4	0.0	17.0	
	(2–4) hours	n	117	119	6	242	
		%	46.8	32.7	37.5	38.4	
	More than 4 h	n	82	189	10	281	
		%	32.8	51.9	62.5	44.6	
Age (Years)	Mean ± SD		20.73 ± 1.33	20.69 ± 1.4	20.68 ± 0.99	20.69 ± 1.4	0.982^(2)^
	Range		18–24	18–24	18–23	18–24	

Bold values is significant* P* value

*SD* standard deviation

^*^statistically significant at *P* ≤ 0.05

^(1)^Chi Square test ^(2)^ One Way ANNOVA test

**Fig. 1:**
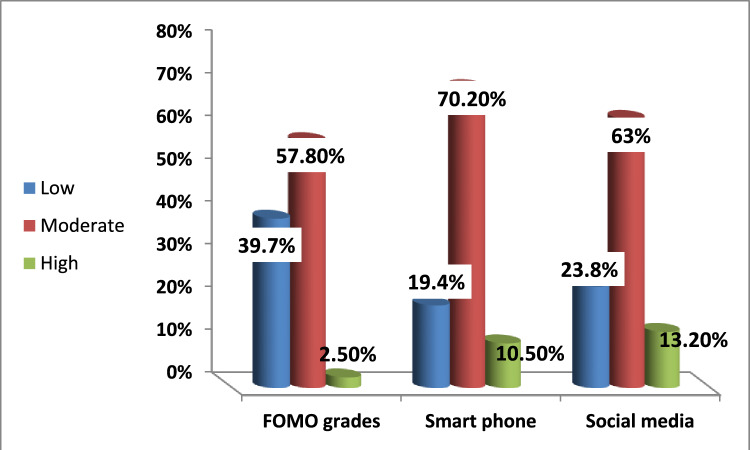
FOMO, smart phone and social media addiction grades among medical students

The prevalence of FOMO among medical students is 57.8% moderate and 2.5% high grade (Fig. 1). The analysis of various sociodemographic factors related to FOMO shows that several variables do not exhibit a significant effect on FOMO levels. Gender, with males and females, residence as urban and rural residents have nearly similar distributions of low, moderate, and high FOMO (*p* = 0.688, 0.912), respectively. Similarly, the academic year, from first to fifth, does not show significant variation in FOMO levels (*p* = 0.333). Participation in hobbies or sports also does not significantly affect FOMO, as those with and without hobbies or sports show similar FOMO distributions (*p* = 0.486). In addition, age does not significantly affect FOMO levels (*p* = 0.982). The data on the number of hours spent on social media reveals a significant association with FOMO levels (*p* < 0.001). Specifically, 62.5% of those with a high FOMO score were spending more than 4 h per day on social media (Table 1).

The prevalence of smartphone addiction among medical students is 70.2% moderate and 10.4% high addiction grade (Fig. 1). The analysis of smartphone addiction across various sociodemographic factors reveals that several aspects do not significantly affect smartphone addiction levels except gender, which shows a notable association (*p* < 0.001), with females experiencing higher levels of high smartphone addiction (83.3%) compared to males (16.7%). However, residence (urban vs. rural), academic year across different years, participation in hobbies or sports, the number of hours spent on social media per day, and age of the studied participants do not significantly influence smartphone addiction levels (*p* = 0.324, 0.168, 0.497, 0.221, 0.855), respectively (Table 2).

**Table 2:** Relationship between sociodemographic data and smartphone addiction levels among medical students

Sociodemographic data			Smartphone Addiction Levels			Total N = 630	*P* value
			Low n = 122(19.4%)	Moderate n = 442(70.2%)	High n = 66(10.4%)		
Gender	Male	n	46	197	11	253	<0.001*^(1)^
		%	37.7	44.6	16.7	40.3	
	Female	n	76	245	55	376	
		%	62.3	55.4	83.3	59.7	
Residence	Urban	n	67	263	33	363	0.324^(1)^
		%	54.9	59.5	50	57.6	
	Rural	n	55	179	33	266	
		%	45.1	40.5	50	42.4	
Academic year	First	n	18	60	6	84	0.168^(1)^
		%	14.7	13.6	9.1	13.3	
	Second	n	23	95	11	129	
		%	18.9	21.5	16.7	20.5	
	Third	n	47	204	37	287	
		%	38.5	46.2	56.1	45.7	
	Fourth	n	18	55	9	82	
		%	14.8	12.4	13.6	13.0	
	Fifth	n	16	28	3	47	
		%	13.1	6.3	4.5	7.5	
Hobbies and sports	No	n	53	166	25	244	0.497^(1)^
		%	43.4	37.6	37.9	38.7	
	Yes	n	69	276	41	385	
		%	56.6	62.4	62.1	61.3	
Number of hours using social media per day	Less than 2 h	n	28	68	11	107	0.221^(1)^
		%	23.0	15.4	16.7	17	
	(2–4) hours	n	48	172	22	242	
		%	39.3	38.9	33.3	38.4	
	More than 4 h	n	46	202	33	281	
		%	37.7	45.7	50	44.6	
Age (Years)	Mean ± SD		20.73 ± 1.206	20.7 ± 1.15	20.63 ± 1.009	20.7 ± 1.146	0.855^(2)^
	Range		18–24	18–24	18–23	18–24	

Bold values is significant* P* value

*SD* standard deviation

^*^statistically significant at *P* ≤ 0.05

^(1)^Chi Square test ^(2)^ One Way ANNOVA test

Regarding social media addiction, 63 and 13.2% are moderate and high addicts (Fig. 1). The analysis of social media addiction across different factors indicates several significant associations; gender shows a significant relationship (*p* = 0.008), with females experiencing higher levels of high social media addiction (71.1%) compared to males (28.9%). Residence also has a significant effect (*p* = 0.025), where urban residents report higher levels of high addiction (50.6%) compared to their rural participants (49.4%). The academic year shows significant variation (*p* = 0.041), with third-year students exhibiting the highest levels of high social media addiction (49.4%), while fifth-year students show the lowest (1.2%). However, participation in hobbies or sports, the number of hours spent on social media per day, and age do not significantly affect social media addiction levels (*p* = 0.142, 0.448, 0.074), respectively (Table 3).

**Table 3:** Relationship between sociodemographic data and social media addiction levels among medical students

Sociodemographic data			Social Media Addiction Levels			Total N = 630	*P* value
			Low n = 150(23.8%)	Moderate n = 397(63%)	High n = 83(13.2%)		
Gender	Male	n	74	156	24	254	0.008*^(1)^
		%	49.3	39.3	28.9	40.3	
	Female	n	76	241	59	375	
		%	50.7	60.7	71.1	59.7	
Residence	Urban	n	76	245	42	363	0.025*^(1)^
		%	50.7	61.7	50.6	57.6	
	Rural	n	74	152	41	266	
		%	49.3	38.3	49.4	42.4	
Academic year	First	n	21	55	8	84	0.041*^(1)^
		%	14.0	13.9	9.6	13.3	
	Second	n	32	79	18	129	
		%	21.3	19.9	21.7	20.5	
	Third	n	57	190	41	287	
		%	38.0	47.9	49.4	45.7	
	Fourth	n	21	46	15	82	
		%	14.0	11.6	18.1	13.0	
	Fifth	n	19	27	1	47	
		%	12.7	6.8	1.2	7.5	
Hobbies and sports	No	n	63	143	38	244	0.142^(1)^
		%	42.0	36.0	45.8	38.7	
	Yes	n	87	254	45	385	
		%	58.0	64.0	54.2	61.3	
Number of hours using social media per day	Less than 2 h	n	33	60	14	107	0.448^(1)^
		%	22.0	15.1	16.9	17.0	
	(2–4) hours	n	55	155	32	241	
		%	36.7	39.0	38.6	38.4	
	More than 4 h	n	62	182	37	281	
		%	41.3	45.8	44.6	44.6	
Age (Years)	Mean ± SD		20.88 ± 1.31	20.63 ± 1.118	20.68 ± 0.915	20.7 ± 1.146	0.074^(2)^
	Range		18–24	18–24	19–23	18–24	

Bold values is significant* P* value

*SD* standard deviation

^*^statistically significant at *P* ≤ 0.05

^(1)^Chi Square test ^(2)^ One Way ANNOVA test

**Fig. 2:**
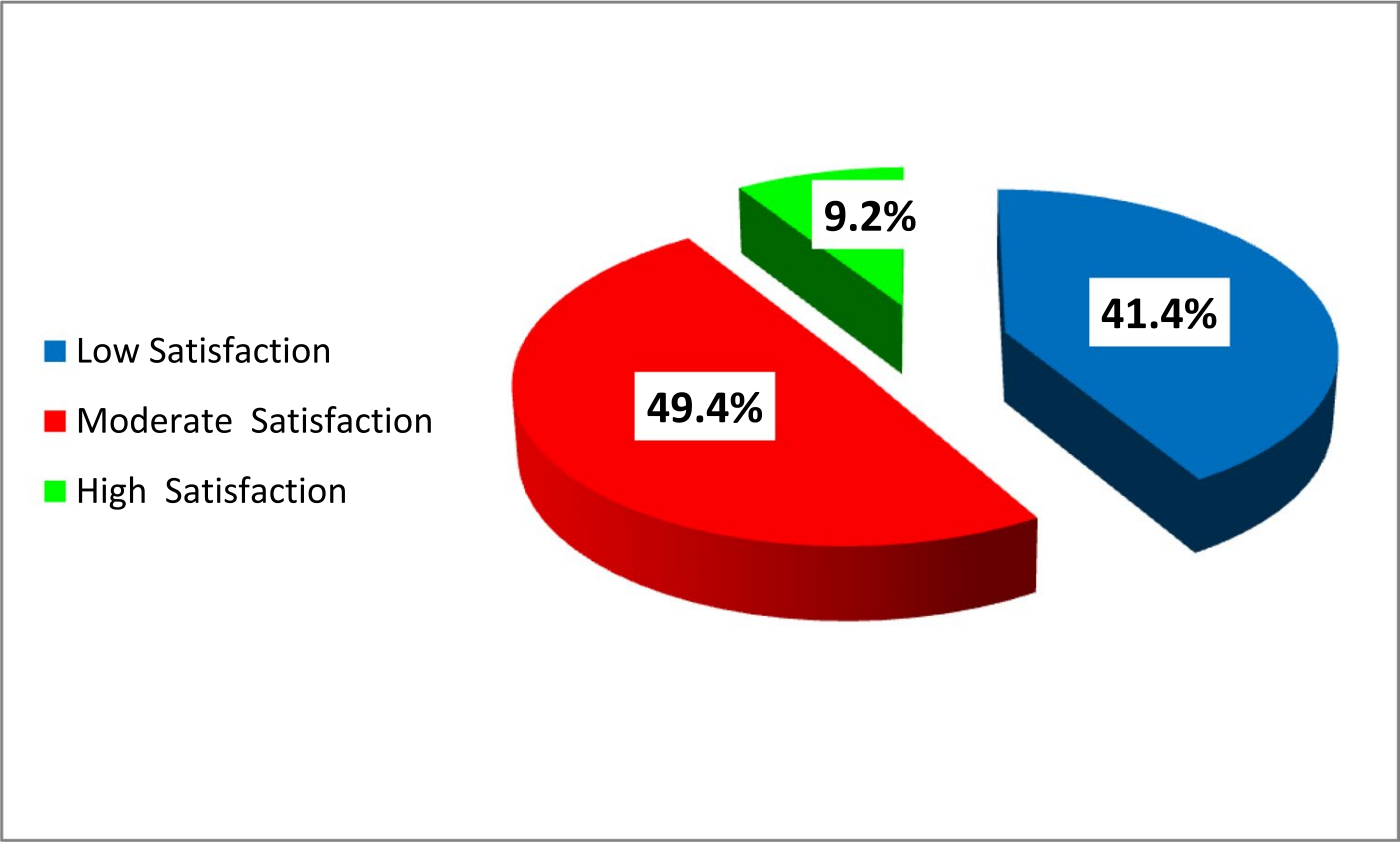
Satisfaction level among medical students

Figure 2 showed the satisfaction level among medical students. About half of the students (49.4%) were moderately satisfied with their lives, and 41.4% showed a low satisfaction level. Meanwhile, 9.2% were highly satisfied.

The Pearson correlation matrix reveals that FOMO is significantly positively correlated with both smartphone addiction (*p* < 0.001, rs = 0.393) and social media addiction (*p* < 0.001, rs = 0.304). FOMO shows a small but significant negative correlation with life satisfaction (*p* = 0.027, rs = −0.064). Smartphone addiction has a moderate positive correlation with social media addiction (*p* < 0.001, rs = 0.542), but neither smartphone addiction (*p* = 0.105, rs = 0.065) nor social media addiction (*p* = 0.392, rs = −0.011) significantly impacts life satisfaction (Table 4).

**Table 4:** Pearson correlation between FOMO, smartphone addiction, social media addiction scales and life satisfaction scale

	Pearson Correlation	FOMO	Smartphone addiction	Social media addiction	Life satisfaction
FOMO	rs	–	–	–	–
	*P* value	–	–	–	–
Smartphone addiction	rs	0.393	–	–	–
	*P* value	0.000*	–	–	–
Social media addiction	rs	0.304	0.542	–	–
	*P* value	0.000*	0.000*	–	–
Life satisfaction	rs	−0.064	0.065	−0.011	–
	*P* value	0.026*	0.105	0.392	–

Bold values is significant* P* value

*FOMO* fear of missing out, *rs* Correlation coefficient

^*^Statistically significant at *P* ≤ 0.05

There is a significant association between FOMO levels and life satisfaction (*p* < 0.001), with individuals experiencing low FOMO showing higher life satisfaction, as evidenced by 43.1% with high life satisfaction having low FOMO scores. In contrast, smartphone addiction (*p* = 0.232) and social media addiction (*p* = 0.220) do not show significant associations with life satisfaction (Table 5).

**Table 5:** Relationship between FOMO, smartphone addiction, social media addiction scales and life satisfaction scale

			Life satisfaction			Total N = 630	*P* value
			Low n = 261(41.4%)	Moderate n = 311(49.4%)	High n = 58(9.2%)		
FOMO	Low	n	89	136	25	250	<0.001^*(1)^
		%	34.1	43.7	43.1	39.7	
	Moderate	n	166	171	27	364	
		%	63.6	55	46.6	57.8	
	High	n	6	4	6	16	
		%	2.3	1.3	10.3	2.5	
Smart phone addiction	Low	n	53	54	15	122	0.232^(1)^
		%	20.3	17.4	25.9	19.4	
	Moderate	n	186	217	39	442	
		%	71.3	69.8	67.2	70.2	
	High	n	22	40	4	66	
		%	8.4	12.9	6.9	10.5	
Social media addiction	Low	n	54	76	20	150	0.220^(1)^
		%	20.7	24.4	34.5	23.8	
	Moderate	n	168	197	32	397	
		%	64.4	63.3	55.2	63	
	High	n	39	38	6	83	
		%	14.9	12.2	10.3	13.2	

Bold values is significant* P* value

^*^Statistically significant at *P* < 0.05

^(1)^Chi Square test

## Discussion

It is critical to know what threats the internet and social media can bring by understanding the variables linked to problematic social media use. In an attempt to adjust to the advancement of technology applications in aspects of the human’s life and improve the general lifestyle. Especially with the growing dependence on artificial intelligence and information technology among medical students who are frequent users of social media applications and who will eventually hold leadership positions. There are few studies examining this relationship among Egyptian medical students.

Thus, this study aims to assess the prevalence of the fear of missing out, smartphone addiction, and social networking addiction; the relationship between them; and also their relationship with life satisfaction. A cross-sectional study design was employed, involving a survey questionnaire administered to a sample of medical students at Tanta University who were selected randomly.

### Prevalence of social media, smartphone addiction, and FOMO

Our study revealed that 63% of medical students exhibited a moderate level of social media addiction. This finding is consistent with a Saudi Arabian study, which reported the prevalence of social media addiction was 55.2% among medical students [11]. Furthermore, our results showed that 70.2% of participants were moderate smartphone addicts, aligning with a recent study in Bangladesh that found 61.4% of young adults in 2022 were addicted to their smartphones. The unemployment rates, technology addiction, inadequate education, conscious parenting, and ‘conscious consumerism’ in the study area and countries like Bangladesh may have contributed to the increased use of social media and smartphones compared to other regions of the world [18].

Such a high prevalence of both social media and smartphone addiction among the participants is in line with smartphone usage rates in Southeast Asia compared to other regions of the world [19]. Medical students are more likely to use smartphones as a coping mechanism to alleviate stress. This reliance on smartphones may be exacerbated by a lack of interpersonal communication, as literature suggests that individuals who experience social isolation or inadequate face-to-face interaction may be more inclined to use smartphones as a substitute for human connection [20].

Additionally, some medical students have developed a phone addiction as a result of their excessive use over the past few years, which has been exacerbated by the COVID-19 pandemic, the challenges of online learning, and the extended periods of time spent at home away from the social pressures of the classroom [21]. Social media is used for medical education by 86.1%, with YouTube, Instagram, and Facebook being the most used [22].

In our study, more than one half of the subjects (57.8%) had a moderate level of FOMO. Along with that, a study conducted in Indonesia found that 50.7% of subjects had a moderate level of FOMO [23]. This suggests that college students who have unfulfilled psychological needs, like a sense of belonging, are more likely to experience FOMO, which is likely to result in a poor social life as well as decreased performance and productivity [24]. A high grade of FOMO score (48.8%) was detected in a study made in Pakistan in 2023 among medical students compared to 2.5% in our study [13]. The severe grade of FOMO in the Pakistani study may be due to using different FOMO scales.

The high prevalence of FOMO can be interpreted in the light of the unique needs that medical students face. The intense study load, the continued search for updates, and the desire to establish social connections in a highly competitive environment may be the driving force. Additionally, the university environment encourages the frequent use of digital devices, such as smartphones and computers, for learning, interaction with classmates, and staying connected to the global health scenario [25].

### Relationships with sociodemographic characters

The analysis of various sociodemographic factors related to FOMO shows that the data on the number of hours spent on social media reveals a significant association with FOMO levels. In agreement, FOMO was positively correlated with increased time spent on social media among European students [26]. Similarly, Fumagalli et al. [27] reported the same results in their study.

FOMO can cause individuals to feel the need to constantly monitor the lives and activities of others. This may lead individuals to constantly check their social media platforms, and this constant attachment state and process may weaken individuals’ self-control, contributing to spending more time in digital environments [28].

### Gender

Our study showed no significant gender differences with FOMO scores and was mainly higher among females. In agreement with a study conducted in Indonesia in 2022, there was no significant gender relationship with FOMO [23]. However, there are studies that found a significant gender difference, with some reporting the highest levels of FOMO in females [29] and others in males [24]. Gender disparities in culture could also be a factor. This discrepancy can still be explained by pure coincidence [17].

Regarding smartphone addiction, our study revealed a significant relationship with gender, and 83.3% of females experienced a high grade of smartphone addiction compared to 16.7% of males. There are studies that found a significant gender difference, with some reporting the highest levels of smartphone addiction in females [30] and others in males [31]. Others with no gender difference [32].

The differences may well exist in how males and females use their smartphones. Males are likely to be more interested in the technological aspects of the cell phones, whereas females are more concerned with social interaction [31]. However, it has been reported recently that the gender gap in cellular phone usage has practically vanished, and this is approved in our study: 55.4% of females vs. 44.6% of males were moderate smartphone addicts [33].

Based on our study results, social media addiction was found to have a significant relationship with gender; 71.1% of females experienced a high grade of social media addiction compared to 28.9% of males. This result is in conjunction with a study conducted by Li et al. [34], stating that being a female, a student, and having fewer social supports increased the severity of internet addiction. Another study done in Indonesia also supported the same evidence, where 34.6% of females experienced internet addiction while no male experienced internet addiction [35]. However, this result wasn’t in conjunction with a Chinese study, which stated that high internet addiction (2.53%) was found in males compared to 0.63% among females [36]. Males are usually addicted to online games, while females often get addicted to chatting and online shopping. This may be because females in their adolescence were more concerned about social evaluation and more anxious about rejection in peer groups [37].

### Residence

The analysis of social media addiction across different demographic factors indicates several significant associations; residence has a significant effect, where urban residents report higher levels of high addiction compared to their rural participants.

The results can be compared to those of Zewde et al. [38] in 2022, who reported a higher significant difference among urban students. While residence does not significantly influence addiction levels in an Indian study in 2021, urban students are still higher than rural students [39]. Again, in Czechoslovakia in 2022, residence does not significantly influence addiction levels and is also slightly higher in urban areas [40].

Whether residence is significant or not, urban students are still higher than rural ones. Urban areas are more internet accessible, and urban individuals make greater use of the internet, which may explain the higher rates of addictive behavior in urban areas [41].

### Academic year

Regarding the academic year, it shows a significant relationship with social media addiction, with third-year students exhibiting the highest levels of high social media addiction, while fifth-year students show the lowest. This is consistent with the findings of Arzani-Birgani et al. [42], who concluded that age is inversely related to internet addiction. Regarding the new younger generations, their greater susceptibility can be explained by an increase in individualism, lower sociability, and enculturation [43].

### Age

Regarding age, our study didn’t show a significant difference in social media addiction, smartphone addiction, and FOMO scores. This is supported by a study done in China [12] but is opposed by other studies that found a negative correlation between age and both FOMO and social media engagement [23, 34]. The wider age group of the latter two cited studies possibly explains why they had results that differ from our study; younger individuals are more likely to use social networking services frequently and thus are more prone to its effects, including FOMO, and since our participants comprise a narrow age group and are limited to a younger age, it may not show the same variability as the mentioned studies.

### Participation in hobbies

Contrary to expectations, our series demonstrated that participation in hobbies or sports does not significantly influence social media, smartphone addiction, and FOMO scores, with those participating in hobbies having a higher addiction and FOMO score. Sports and hobbies can be one of the protective factors against technology addiction, and this is evident during childhood periods, according to a Korean study in 2022 [44]. However, its role in university age is doubtful.

### Relationship between FOMO, smartphone addiction, social media addiction scales, and life satisfaction scale

In our study, FOMO had a significantly positive correlation with social media addiction. This finding was supported by Li et al.’s [45] study. Additionally, Monteiro et al. [46] demonstrated that due to students’ increasing fear of missing out, they became more preoccupied with social media activities.

The reported positive relationship between FOMO and addiction to social media became evident as social media use is prevalent and contributes significantly to the lifestyle of the youth. Individuals who suffer from FOMO constantly follow what others are doing, and this caused them to use social media regularly. This was evident as the use of social media was convenient to get the latest information and meet their social needs. One can easily feel inferior if they feel left out of something, which can be particularly true for the youth who are still seeking to identify their place in society [47].

Thus, people who experienced high levels of FOMO tended to use Facebook more frequently right after waking up, right before bed, and right before meals. Students with high FOMO levels expressed conflicting emotions about social media and were more inclined to use Facebook in class [1].

Additionally, it has been proposed that FOMO is a direct predictor of problematic social media use or a mediator in the relationships between psychopathological symptoms and negative outcomes arising from use of social networking sites, given that the desire or need to be continuously connected with others is easily satisfied by using these sites. As a result, people may attempt to control their FOMO by using social media excessively because they think it might help them control their fear of being left out [48].

Smartphone addiction had a significant moderate positive correlation with social media addiction in our study. In agreement, the results of a study done in Turkey showed that the first cause of social media addiction among adolescents was smartphone addiction [49]. However, addiction patterns of smartphones can be claimed to originate from applications such as calling, messaging, or social networking sites on smartphones [46].

This outcome is a reflection of the widespread use of digital gadgets worldwide. Smartphones offer high portability simultaneously with web availability, which has prompted a considerable increment in the number of people using social media, particularly the ‘Twenty to thirty-year-olds.’ The overutilization of smartphones and social media has resulted in a few issues affecting people’s mental conditions [50].

The current study shows that FOMO has a significant negative correlation with life satisfaction. Many studies in the related literature show a negative relationship between FOMO and satisfaction with life. This finding indicates that as the individual’s FOMO level increases, his/her life satisfaction decreases [42]. In accordance, a Turkish study in 2021 showed higher overall levels of FOMO were associated with lower levels of need satisfaction, general mood, and overall life satisfaction [1].

Throughout university years, students live independently and are apart from their families, but they nevertheless keep in touch with them virtually. When a person’s basic need for social relationships is satisfied, they become less reliant on virtual settings. As a result, his or her level of FOMO drops, and life happiness rises. The urgent use of social media to facilitate maintaining interpersonal relationships, meeting new friends, and expanding students’ social environments suggests that students’ needs are met, which further improves their satisfaction towards life [51].

## Limitations

As this is a cross-sectional study, there are limits to the conclusions that can be drawn. Because the data used for the present study was collected through one-time surveys, they reflect snapshots of individuals in time.

The causal direction of these relationships cannot be determined, so it is unclear if social media use causes more FOMO or if FOMO leads to experiencing social media addiction (which caused which).

This research study was based on participants’ subjective feelings, self-reported data, and fears of missing out at any given point in time, but we didn’t have an objective assessment of whether or not they were missing out on anything. A more reliable questionnaire is needed that excludes the psychological problems that may result in FOMO and assesses the activities and hobbies that may help in reducing FOMO or act as protective factors.

In the present study, some personality characteristics of late adolescents were not taken into consideration as risk factors. Next studies can focus on personality variables to find out risk factors for social media addiction. A large-scale study can be held to find out the profile of adolescents social media usage and risk and protective factors. This study didn’t include the variety of internet use motives by students, so the type of internet addiction couldn’t be made.

## Future research

More research is needed to understand what components of health are most influenced by FOMO and how health may be improved through interventions to reduce FOMO. Longitudinal studies can be carried out to explain how experiencing FOMO influences behaviors and health over time and to establish directional relationships between FOMO, social media use, and health. Future research would be conducted on fear of missing out to know about other potential factors of fear of missing out that would explain both the feelings and outcomes among university students. More comparative studies using different study tools should be conducted on the fear of missing out between the school, college, and university populations that would highlight more diversity in results.

## Conclusion

The prevalence of FOMO among medical students was an alarming sign. Also, social media and smartphone addiction have been becoming issues of concern. The findings of the study showed that FOMO is significantly positively correlated with both smartphone addiction and social media addiction. Smartphone addiction has a moderate positive correlation with social media addiction. FOMO shows a small but significant negative correlation with life satisfaction.

## Recommendations

- Elective education program specially designed to mitigate problematic social media use. By designing an interactive course or workshop, focusing on increasing awareness of social media issues. Also, involve students in social activities and elective activities inside the faculty.
- It is important that positive social, emotional, and physical values are addressed through curriculums studied by medical students.
- Improving the design of classroom activities so that they fully utilize the benefits of digital applications without sacrificing the crucial role of physical classroom activities.
- Psychological screening is also suggested to detect students who struggle with self-esteem that is related to various life aspects such as social, family, financial, and physical.
- Counselling unit at the faculty to acknowledge the importance of social involvement and life happiness.

## Data Availability

The datasets generated and analyzed during the current study are not publicly available. However, the datasets are available from the corresponding author on a reasonable request.
